# Serological examination for clinical cross-reactivity between salmon roe and pollock roe in patients with a salmon roe allergy

**DOI:** 10.20407/fmj.2021-004

**Published:** 2021-08-20

**Authors:** Kaoru Okamoto, Yoichi Nakajima, Tetsushi Yoshikawa, Yasuto Kondo

**Affiliations:** Department of Pediatrics, School of Medicine, Fujita Health University, Toyoake, Aichi, Japan

**Keywords:** Food allergy, Fish roe, Cross-reactivity, ELISA, Immunoblot

## Abstract

**Objectives:**

Fish roe is a common allergen in Japan. We have previously reported that although immunoglobulin (IgE) from patients with salmon roe (SR) or pollock roe (PR) allergies cross-react, 70% of patients with SR allergy can consume PR without developing any symptoms. However, a correlation between clinical cross-reactivity and serological cross-reactivity remains to be demonstrated.

**Methods:**

Serum samples were collected from 15 patients with SR allergy who had consumed cooked PR previously. Among these volunteers, four had experienced immediate symptoms after consuming cooked PR, while the others had exhibited no symptoms of PR allergy. A competitive enzyme-linked immunosorbent assay (ELISA) was performed to analyze the serological cross-reactivity with SR and PR. Immunoblotting inhibition assays were performed using serum samples that had been pre-incubated with SR or PR extracts.

**Results:**

In ELISAs, binding to SR was inhibited by >50% when the serum samples from patients with both SR and PR allergies were pre-incubated with PR extract (*p*=0.0256). In immunoblots, pre-incubation of serum samples with PR extract inhibited detection of the 16-kDa protein, which likely corresponds to the major SR allergen beta' component, significantly more for samples from patients with both SR and PR allergies (100%) than for samples from those with only an SR allergy (18.2%) (*p*=0.011).

**Conclusions:**

The superior competitive binding of the sera from patients with both SR and PR allergies to PR compared with that to SR may induce clinical cross-reactivity between SR and PR.

## Introduction

In Japan, certain types of fish roe are common in various culinary dishes, such as in sushi, salad, and pasta. The prevalence of allergy to fish roe, which is also on the rise in Western countries,^1,2^ has increased there, and it is reported to be the second most common food allergy in Japan among those aged 1 to 6 years.^3^ In particular, salmon roe (SR) allergy, which sometimes causes severe immediate symptoms, is “recommended for allergic labeling” on food by the food sanitation law in Japan. There have also been some reports of allergic symptoms following the consumption of pollock roe (PR) or capelin roe, which are commonly eaten in Japan. Unlike SR, which is typically eaten raw, PR and capelin roe are usually eaten after being cooked. Shimizu et al. reported that the major SR allergen is the beta'-component (β'-c), which is a degraded fragment of vitellogenin.^4^ The β'-c of SR is registered as a major allergen, “Onc k 5”, on the official allergen list published by the World Health Organization. This allergen has also been reported to have common antigenicity with other types of fish roe; however, common serological allergenicity does not always cause clinical cross-reactivity.^5,6^ Given the issue of potential cross-reactivity, patients with SR allergy might need to avoid the consumption of other roe, such as PR and capelin, as well. We reported in a previous study that the sera of patients with SR allergy showed immunoglobulin (Ig)E cross-reactivity among multiple types of fish roe, including PR.^7^ However, many patients who are allergic to SR can eat PR or capelin roe without experiencing immediate symptoms. Another study reported that the proportion of PR allergy among Japanese patients with SR allergy is 25%, and the proportion of capelin roe allergy in this population is 11%.^8^ Thus, although 75% of patients with SR allergies can eat PR safely, the difference in allergens between patients with multiple fish roe allergies and patients allergic to only SR remains unknown. It is important to address this issue, predominately to improve the quality of life for patients with SR allergies but also to resolve the impact of this common allergy on school lunch restrictions. Makita et al. reported that the ratio of PR-specific IgE/SR-specific IgE is more useful for predicting a positive result to a heated PR oral food challenge compared with the level of PR-specific IgE alone^9^; to the best of our knowledge, however, no other previous studies have investigated methods of distinguishing between patients with and without PR allergy.

In the present study, we examined the cross-reactivity between SR and PR using enzyme-linked immunosorbent assay (ELISA) and immunoblot inhibition methods on serum samples from patients with a known SR allergy who had ever consumed PR, and we assessed the association between actual symptoms *in vivo* and serum reactivity *in vitro*.

## Methods

### Human sera and diagnosis of fish roe allergy

SR allergy was diagnosed on the basis of at least one convincing report or positive result from a skin-prick test using SR extract and a positive result (>0.70 IU/ml) from a test for salmon roe-specific IgE conducted using the ImmunoCAP system (ThermoFisher Scientific, Waltham, MA, USA). PR allergy was diagnosed in the same manner as SR allergy. All collected samples were stored at –20°C until use. Ethical approval was obtained from the Fujita Health University Ethics Committee in May 2015 (reference number: HM19-394). We obtained informed consent from all participants or their parents.

### Extraction

Fish roe extracts were produced as described in our previous study.^7^ Briefly, we pulverized raw fish roe from salmon (*Oncorhynchus kisutch*) or pollock (*Theragra chalcogramma*), centrifuged 5 g of the product in 15 ml of 1 M potassium chloride–phosphate-buffered saline (PBS) in a 50-ml sterile centrifuge tube at 15,000×*g* for 10 min, and placed the resulting supernatant in a cold room (4°C) overnight. After centrifuging the tubes, we dialyzed the resulting supernatants against distilled water with a dialysis tube (cut-off: molecular weight of 6000–8000) in a cold room overnight. We filtered the resulting concentrates using a Millex-HP 0.45-μm filter (Merck Millipore Ltd, Burlington, MA, USA) and stored them at –20°C. We determined the protein concentration of each extract by using a bicinchoninic acid protein assay (ThermoFisher Scientific).

### Enzyme-linked immunosorbent assay (ELISA)

The main elements of the applied ELISA and ELISA inhibition procedures were based on those described in our previous study.^7^ Here, we diluted the extracts with a PBS buffer to a concentration of 1 μg/ml for the SR extract and 10 μg/ml for the PR extract. We placed samples (100 μl/well) in each Nunc-Immuno Plate (ThermoFisher Scientific) and incubated them for 1.5 h at 25°C. We then discarded the samples, added SuperBlock Blocking Buffer in PBS (150 μl/well; ThermoFisher Scientific), and stored the plate at 25°C for 1 h. After discarding the buffer, we washed each well three times with 200 μl of PBS-Tween. We then added 100 μl/well of patient serum diluted with PBS-Tween (1:20) to each well and stored the plate overnight at 4°C. After washing each well with PBS-Tween and discarding the sera, we added Biotinylated Anti-Human IgE (epsilon) Antibody (SeraCare, Milford, MA, USA) diluted with PBS-Tween (1:1000, 100 μl/well) and incubated the plate at 25°C for 1 h. We then washed each well with PBS-Tween, added horseradish peroxidase-conjugated streptavidin (Proteintech Group, Inc., Rosemont, IL, USA) diluted with PBS-Tween (1:5000, 100 μl/well), and incubated the plate at 25°C for 1 h. After washing each well, we incubated the cells with 3,3',5,5'-Tetramethylbenzide Liquid (MP Biomedicals, Inc. Irvine, CA, USA) for 30 min under a light shield. The reaction was stopped with 100 μl/well of 1 N hydrochloric acid, and the results were measured with a Wako SUNRISE Rainbow-RC-R (WAKENYAKU Co., Ltd, Kyoto, Japan).

For the inhibition ELISAs, we pre-incubated the patient sera with one of four different concentrations (0, 10, 100, and 1000 μg/ml) of SR or PR extract as an inhibitor before adding the samples to an ELISA plate pre-coated with SR or PR extract. The subsequent procedure was the same as that used for the ELISA described above. We defined an inhibition of more than 50% with heterogeneous fish roe extract as efficient inhibition, and we considered that there was serological cross-reactivity between both types of fish roe in such cases.

### Immunoblotting

We performed sodium dodecyl sulfate-polyacrylamide gel electrophoresis (SDS-PAGE) in a NuPAGE 4%–12% Bris-Tris Precast Gel (ThermoFisher Scientific). Proteins were separated at 200 V for 1 h. After this procedure, we transferred the proteins to Immobilon-P Transfer Membranes (Merck Millipore Ltd) as previously reported.^10^ To detect bound IgE, we used a phosphatase-labeled goat anti-human IgE (epsilon) antibody (SeraCare) diluted 1:2000 and developed the blot with BCIP/NBT solution (SeraCare) at 25°C.

After protein transfer, we stained the Immobilon-P membranes with Amido Black 10B (FUJIFILM Wako Pure Chemical Corporation, Osaka, Japan) in 50% methanol and 10% acetic acid, then de-stained them with 40% methanol and 10% acetic acid, and air dried them.

### Immunoblot inhibition

After the SR or PR proteins were transferred to the Immobilon-P membrane, we added the serum samples of patients with SR allergy that had been pre-incubated with solutions containing SR or PR extracts (0, 0.6, or 3.0 mg) as inhibitors. To detect bound IgE, we used the same procedure as described above for immunoblotting.

### Statistical analysis

For statistical comparisons between the results of patients with coexisting PR and SR allergies and patients with only an SR allergy, we used a Fisher’s exact test. A *p*-value of <0.05 was considered to indicate a significant difference. We performed these statistical analyses with EZR (Saitama Medical Center, Jichi Medical University, Saitama, Japan), which is a graphical user interface for R (The R Foundation for Statistical Computing, Vienna, Austria). More precisely, it is a modified version of the R commander designed to include the statistical functions frequently used in biostatistics.^11^

## Results

This study enrolled 15 patients (male:female=8:7, age range: 2–12 years) who were allergic to SR and had consumed PR previously (Table 1). Four of these patients had experienced immediate symptoms following the consumption of cooked PR: two experienced anaphylaxes (vomiting and systemic urticaria), one had a history of facial urticaria, and one experienced oral swelling and a scratchy throat.

### Association between PR allergy and the reaction pattern of ELISA inhibition

The results of the ELISA inhibition for each fish roe type with the maximum inhibition rate (%) induced by pre-incubation with a heterogeneous fish roe extract are shown in Table 2. The binding of IgE from the serum samples of patients with a PR allergy to the SR extract was inhibited by >50% following pre-incubation of these samples with PR extract. However, the binding of IgE from the serum samples of patients with an exclusive SR allergy, except for those of three patients (N-1, N-2, N-3), was not effectively inhibited by pre-incubation of the serum samples with PR extract (*p*=0.0256, Fisher’s exact test). There was efficient inhibition of IgE binding to the PR extract caused by pre-incubation of the serum sample from two (50%) patients with PR allergy and four (36.4%) patients with an exclusive SR allergy with SR extract.

On the basis of these results, the data from the 15 patients could be divided into four patterns: (1) equivalent competitive inhibition (n=3; P-1, P-2 and N-1) ; (2) PR superior competitive inhibition (n=4; P-3, P-4, N-2 and N-3); (3) SR superior competitive inhibition (n=3; N-4, N-5 and N-6); and (4) no significant inhibition to each other (n=5; N-7, N-8, N-9, N-10 and N-11).

### Electrophoresis using SDS-PAGE and immunoblot

**Figure 1:**
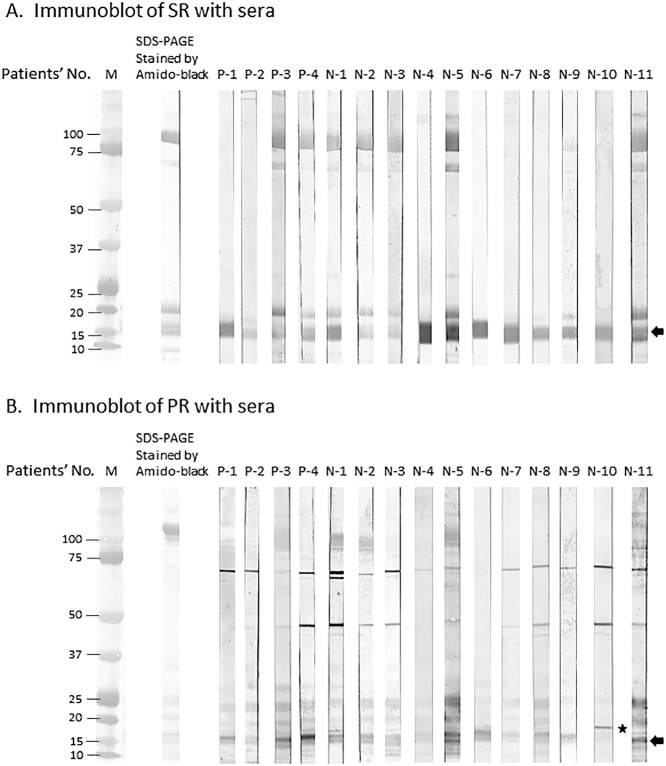
SDS-PAGE gels stained with Amido-black and immunoblots of SR and PR. (A) SDS-PAGE gel stained using Amido-black and immunoblot of SR. Arrow indicates the 16–18-kDa protein on the SR immunoblot membrane. (B) SDS-PAGE gel stained using Amido-black and immunoblot of PR. Arrow indicates the 16-kDa protein on the PR immunoblot membrane to which serum samples from almost all patients bound, except for patient N-10, whose serum IgE bound to the 18-kDa protein (star). SDS-PAGE, sodium dodecyl (lauryl) sulfate-polyacrylamide gel electrophoresis; SR, salmon roe; PR, pollock roe; IgE, immunoglobulin E.

Figure 1 shows the results of protein staining and immunoblotting using SDS-PAGE. On the SR immunoblot membranes, there were bands with the three following molecular weights: 16–18 kDa (15 patients; 100%), 20 kDa (7 patients; 46.6%), and/or 75 kDa (5 patients; 33.3%). Among these three bands on the SR immunoblot membranes, the 20-kDa band and 75-kDa band were considered to be non-specific bands because they were not inhibited by pre-incubation of the serum samples with SR extract. The PR immunoblot membranes also had bands with three different molecular weights: 16–18 kDa (15 patients; 100%), 50 kDa (10 patients; 66.7%), and/or 75 kDa (14 patients; 93.3%). The 50-kDa band and 75-kDa band were inhibited in a dose-dependent manner by pre-incubation of the serum samples with SR extract, and their patterns were relatively similar to those of the 16–18-kDa band. Additionally, a recent study reported that the high-molecular-weight band on a PR immunoblot contained the β'-c structure.^12^ Therefore, we focused on the 16–18-kDa band on each fish roe immunoblot membrane, which was presumed to be β'-c.

### Immunoblot inhibition

**Figure 2:**
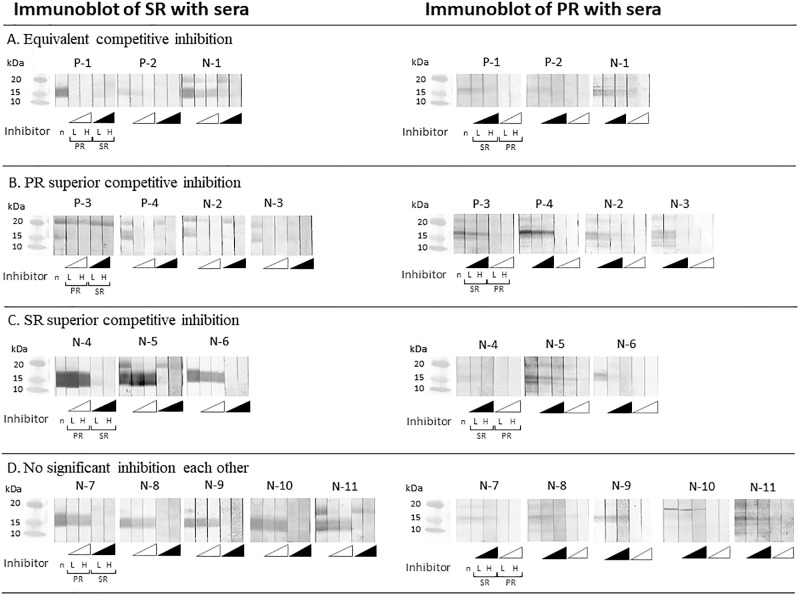
Immunoblot inhibition with heterogeneous fish roe extract, focusing on the 16–18-kDa protein on each fish roe immunoblot membrane. Patterns of competitive inhibition. (A) An example of equivalent competitive inhibition. Serum samples from two patients (P-1 and P-2) with coexisting PR and SR allergies were pre-incubated with heterogeneous fish roe extract. (B) An example of PR superior competitive inhibition. Serum samples from two patients (P-3 and P-4) with coexisting PR and SR allergies were pre-incubated with heterogeneous fish roe extract. (C) An example of SR superior competitive inhibition. (D) An example of no significant inhibition. In this pattern, neither the 14–18-kDa bands on the SR immunoblot membrane nor the 16-kDa bands or 18-kDa bands on the PR immunoblot membrane were inhibited by pre-incubation with heterogeneous fish roe extracts. The amount of inhibitor is shown in the following manner: n, without inhibitor; L, low amount of inhibitor (0.6 mg); H, high amount of inhibitor (3.0 mg). ELISA, enzyme-linked immunosorbent assay; SR, salmon roe; PR, pollock roe

We observed the inhibition of the 16–18-kDa IgE-binding band following pre-incubation of the serum samples with a heterogeneous fish roe extract. According to the ELISA inhibition results, the immunoblot data of our 15 patients were divided into four patterns (Figure 2).

Displaying equivalent competitive inhibition (Fig. 2A), the 16-kDa bands on both the SR and PR immunoblot membranes were inhibited in a dose-dependent manner by pre-incubation of the serum samples with the other type of fish roe extract when using serum samples from patients with a PR allergy. This band remained on immunoblots of serum from one patient with an exclusive SR allergy (N-1), even when high concentrations of heterogeneous fish roe extracts were used for pre-incubation.

In cases of PR superior competitive inhibition (Fig. 2B), the 16-kDa band on the SR immunoblot membranes was inhibited by pre-incubation with PR extract for the serum samples from all patients, whereas this band remained on the PR immunoblot membranes when using the serum samples of three patients (P-3, P-4, and N-2), even when high concentrations of SR extract were used.

In cases of SR superior competitive inhibition (Fig. 2C), the band on the SR immunoblot membrane covered a wide area, spanning from 14–18 kDa, and was not inhibited by pre-incubation with PR extract for samples from any of the patients. The 16-kDa band on the PR immunoblot membrane was inhibited by the pre-incubation of serum samples from two patients (N-4 and N-6) with SR extract. This band on the PR immunoblot membrane was not inhibited by the pre-incubation of a serum sample from patient N-5 with the SR extract.

In cases of no significant inhibition of each other (Fig. 2D), the bands at 16 kDa or 18 kDa on the SR and PR immunoblot membranes were not inhibited by the pre-incubation of serum samples from any of the patients with either fish roe extract.

As shown in Table 3, the appearance of the ~16-kDa protein on the SR immunoblot was significantly more likely to be inhibited by pre-incubation with PR extract of samples from patients with PR allergy (100%) than by pre-incubation with PR extract of samples from those with an exclusive SR allergy (18.2%) (*p*=0.011, Fisher’s exact test).

## Discussion

The existence of common antigenicity between SR and PR was first reported by Kondo et al.^7^ Our group previously found that approximately 25% of patients with SR allergy experienced immediate symptoms after eating cooked PR.^8^ Makita et al. determined that the ratio of PR-specific IgE/SR-specific IgE is associated with a positive reaction to cooked PR, and the optimal cut-off is 0.47.^9^ However, among the patients with both SR and PR allergies in our study, two (50%) of four patient had a PR-specific IgE/SR-specific IgE ratio of <0.47; therefore, it might be difficult to determine whether a patient could eat PR safely by looking at only SR- and PR-specific IgE.

In the present work, we included patients with SR allergy who had eaten PR at least once. We performed ELISA inhibition and immunoblot inhibition assays with serum samples provided by these participants to verify the serological differences between patients experiencing immediate symptoms following PR intake and those who could eat PR without any symptoms. The ELISA inhibition results indicate that the SR binding by IgE from the sera of patients with coexisting PR and SR allergies was inhibited by more than 50% by pre-incubation with PR extract. Conversely, the SR binding by IgE from the sera of patients with an exclusive SR allergy was not inhibited efficiently (less than 50%) by pre-incubation of the serum with PR extract.

The immunoblot inhibition results show that IgE from the sera of all patients could bind to the 16–18 kDa bands on both the SR and PR immunoblot membranes. Because there is IgE competition between SR and PR at these molecular weights, the protein in this band was presumed to be the β'-c, which Shimizu et al. reported as having cross-reactivity between SR and PR.^4^ The β'-c is constructed from 16-kDa and 18-kDa subunits, which have high structural similarity to each other.^13^ The β'-c also shares highly homogeneous amino acid sequences among certain types of fish roe; we previously confirmed efficient inhibition, using the ELISA inhibition method, with regards to the binding of IgE in a dose-dependent manner among SR, PR, and herring roe.^7^

In the sera from all four patients with coexisting PR and SR allergies (P-1 to P-4), the binding of IgE to the β'-c of SR was strongly inhibited by pre-incubation with PR extract. However, for most of the patients with an exclusive SR allergy, including those for whom the SR-binding ability of their IgE was efficiently inhibited by pre-incubation with PR extract in an ELISA inhibition assay (N-1), the binding of their IgE to the β'-c of SR was not inhibited by pre-incubation with PR extract. These results suggest that the induction of immediate symptoms by both SR and PR intake is a consequence of IgE cross-reactivity with the 16-kDa protein (β'-c), particularly in cases where the PR protein binding by SR-specific IgE is superior to that of the SR protein. Although the ELISA inhibition results for patient N-1 displayed equivalent competitive inhibition, the 16-kDa bands on the SR and PR immunoblots were not inhibited by pre-incubation of the serum with heterologous roe, even at high concentrations. Therefore, a lack of inhibition of the 16-kDa band on each fish roe immunoblot is thought to be important for whether or not the tested patient will be able to eat heated PR without experiencing allergy symptoms.

Based on our results, IgE cross-reactivity to the 16-kDa protein (β'-c) shared between SR and PR, particularly stronger competitive binding by SR-specific IgE to the PR than to the SR protein, could cause allergies to both SR and PR. It will be important to develop a simple screening method for distinguishing patients with an exclusive SR allergy from those with joint SR and PR allergies in a future study.

### Limitations

One of the limitations of our study is that almost none of the participants had ever eaten raw PR. Although it is still unclear if cooking fish roe causes a change in its allergenicity, we analyzed patients who had immediate symptoms after eating cooked PR because PR is typically eaten in a cooked form. Additionally, all the patients in our study were younger than 15 years old, they had not previously eaten raw PR, and they would not agree to the performance of an oral food challenge with raw PR. Only one patient (N-2) confirmed that he could eat cooked PR but had immediate symptoms following the consumption of raw PR. His serological results were similar to those of the other patients with PR allergy: an ELISA with SR protein was efficiently inhibited, and the 16-kDa protein (β'-c) on the immunoblot membrane was competitively inhibited by the pre-incubation of his serum with PR extract. This result indicates that at least part of the PR protein could have an epitope that loses its ability to be bound by IgE after heating. Thus, patient N-3, whose serum had similar a serological characterization to that of patient N-2, may also be prone to immediate symptoms following the intake of raw PR, but we could not confirm this outcome because this patient did not want to undergo an oral food challenge.

Another limitation of our study is that the number of participants is small; further research with a larger number of participants will be necessary to confirm our results clinically. Our difficulty in obtaining additional eligible participants may be partially because of the severity of symptoms caused by SR in patient with SR allergy. Those who have experienced severe immediate symptoms, such as anaphylaxis, caused by the intake of SR are likely to avoid eating other fish roes, including PR; thus, they understandably did not want to confirm that outcome with an oral food challenge. Additionally, given that there are few occasions during childhood in which fish roe is commonly consumed, and it is relatively easy to avoid eating fish roe, the participants did not feel the need to confirm that they could eat PR. However, knowing whether they can safely eat fish roe other than SR may become important to them in adulthood.

## Conclusion

Both SR and PR allergy symptoms were present in participants: (1) whose serum IgE binding of SR proteins on ELISA examination was inhibited by more than 50% following pre-incubation of the serum with PR proteins; and (2) displayed cross-reactivity between SR and PR owing to the β'-c, and the IgE binding to the SR β'-c was strongly inhibited on immunoblotting following pre-incubation of their serum with PR extract. This result may suggest that the superior competitive binding of PR by SR-specific IgE, especially to the β'-c, causes immediate symptoms to occur after consuming not only SR but also PR. Further research on cross-reactivity among several kinds of fish roe that can cause allergy and the inclusion of information regarding sensitization sources will be needed to reveal the significance of β'-c allergenicity. Additional research on whether heat or cooking cause changes in allergenicity is also required because some types of fish roe, such as PR or capelin roe, are typically eaten after they are cooked.

## Figures and Tables

**Table1:** Characteristics and fish roe specific IgE titer

PR allergy	No.	Age (years)	sex	Immediate symptoms by each roe		Total IgE (IU/ml)	Specific IgE titer (UA/ml)		PR sIgE/SR sIgE ratio
				SR (raw)	PR (heated)		SR	PR	
(+)	P-1	2	M	urticaria	dyspnea and urticaria	1393.1	16.3	4.81	0.295
	P-2	10	M	vomiting	urticaria	312	6.97	2.61	0.374
	P-3	7	F	urticaria	swelling of face	168.4	1.55	2.28	1.471
	P-4	10	M	swelling of lip	oral swelling and scratchy throat*	1270.6	11.3	12.0	1.062

(–)	N-1	6	F	vomiting	none	1297	7.75	1.81	0.234
	N-2	8	M	Have not eaten**	none	582	1.54	1.42	0.922
	N-3	9	F	dyspnea with wheezing	none	1493	0.92	0.55	0.598
	N-4	4	M	urticaria	none	2615	122	3.26	0.027
	N-5	8	F	swelling of lip	none	1158	19.4	4.23	0.218
	N-6	12	M	swelling of lip and dyspnea	none	250.5	66.8	3.76	0.056
	N-7	3	F	vomiting	none	1481	8.87	0.82	0.092
	N-8	4	M	swelling of lip	none	120	8.01	0.26	0.032
	N-9	5	M	red eye and dyspnea	none	707	4.41	2.47	0.560
	N-10	6	F	urticaria	none	380	1.34	0.58	0.433
	N-11	12	F	running nose and sore throat	none	1895	11.6	0.2	0.017

SR, salmon roe; PR, pollock roe; IgE, Immunoglobulin E sIgE, specific Immunoglobulin E * Because P-4’s symptoms by intake of PR was subjective, we also confirmed the positive result of skin prick test of PR extract. ** Because N-2 have experienced anaphylaxis by intake of PR raw, the diagnosis of SR allergy was done by result of skin prick test, considering the risk of oral food challenge of SR.

**Table2:** ELISA inhibition

PR allergy	No.	Maximum inhibition rate(%) on ELISA*		Inhibition pattern
		Inhibition of IgE binding to SR by PR	Inhibition of IgE binding to PR by SR	
(+)	P-1	53.4	51.9	A
	P-2	65.7	52.5	A
	P-3	76.0	41.2	B
	P-4	67.8	4.1	B

(–)	N-1	64.1	51.4	A
	N-2	73.7	49.6	B
	N-3	69.1	19.6	B
	N-4	17.6	63.2	C
	N-5	22.6	53.9	C
	N-6	27.0	60.0	C
	N-7	46.4	28.5	D
	N-8	12.0	32.6	D
	N-9	18.8	22.6	D
	N-10	38.2	30.3	D
	N-11	35.8	20.9	D

There were four patterns; A) Equivalent competitive inhibition, B) PR superior competitive inhibition, C) SR superior competitive inhibition, D) No significant inhibition each other. * Inhibition rate more than 50% was defined as efficient inhibition. ELISA, enzyme-linked immunosorbent assay; SR, salmon roe; PR, pollock roe; IgE, Immunoglobulin E

**Table3:** Immunoblot inhibition of 16 kDa protein by heterogenous fish roe protein

PR allergy	No.	ELISA Inhibition pattern	Concerning band weighing 16–18 kDa on immunoblot membrane	
			Inhibition of IgE binding to SR by PR	Inhibition of IgE binding to PR by SR
(+)	P-1	A	○	○
	P-2	A	○	○
	P-3	B	○	●
	P-4	B	○	●

(–)	N-1	A	●	●
	N-2	B	○	●
	N-3	B	○	○
	N-4	C	●	○
	N-5	C	●	●
	N-6	C	●	○
	N-7	D	●	●
	N-8	D	●	●
	N-9	D	●	●
	N-10	D	●	●
	N-11	D	●	●

A *white circle* means that the 16–18 kDa protein is inhibited by heterogenous fish roe extract. A *black circle* means that the 16–18 kDa protein is *not* inhibited by heterogenous fish roe extract. SR, salmon roe; PR, pollock roe
